# Taxonomic revision of the neophyte nightshades (*Solanum*, Solanaceae) in European Russia and the North Caucasus

**DOI:** 10.3897/phytokeys.270.169902

**Published:** 2026-02-05

**Authors:** Alexander P. Sukhorukov, Sandra Knapp, Elena A. Glazkova, Dmitry S. Shilnikov, Maria Kushunina, Alexander N. Sennikov

**Affiliations:** 1 Biological Faculty, Lomonosov Moscow State University, Leninskie Gory 1/12, Moscow, Russia Lomonosov Moscow State University Moscow Russia; 2 Natural History Museum, Cromwell Road, SW7 5BD, London, UK Natural History Museum London United Kingdom; 3 Komarov Botanical Institute, Prof. Popova St. 2, St. Petersburg, Russia Botanical Museum, Finnish Museum of Natural History Helsinki Finland; 4 Perkalsky Dendrological Park of the Komarov Botanical Institute, Pyatigorsk, Russia Komarov Botanical Institute St. Petersburg Russia; 5 Botanical Museum, Finnish Museum of Natural History, Helsinki, Finland Perkalsky Dendrological Park of the Komarov Botanical Institute Pyatigorsk Russia

**Keywords:** Alien plants, distribution, neophytes, taxonomic revision

## Abstract

The alien *Solanum* species in European Russia and the North Caucasus have never been revised. Over the past three decades, several exotic species have been found in various parts of the area, but many of them were identified incorrectly or reported using outdated taxonomy. We detailed the distribution data for these species, verified and corrected published reports, and provided updated diagnostic characters and a new identification key. Here, we report and discuss the records and residence status of nine species: *S. elaeagnifolium*, *S. emulans*, *S. heterodoxum*, *S. nitidibaccatum*, *S. rostratum*, *S. scabrum*, *S. sisymbriifolium*, *S. triflorum*, and *S. villosum*. One additional species, *S. nigrum*, appears to be an archeophyte widely distributed in both regions, except the Arctic zone. *Solanum villosum*, previously treated as several segregate taxa (*S. alatum*, *S. luteum*, *S. transcaucasicum*, *S. zelenetzkii*), was first collected in the early 20^th^ century in the North Caucasus, whereas its collections from European Russia are mostly from the late 20^th^ century. *Solanum rostratum* is naturalized in the plains of the North Caucasus and the Lower Volga Region. It has also been found several times in the forest and forest-steppe zones, with one new record reported here. *Solanum triflorum* is naturalized and rapidly spreading in the Lower Volga and the eastern North Caucasus; it is recorded here as a new casual alien in Kazakhstan. *Solanum nitidibaccatum* is reported for the first time from several administrative units and is considered naturalized at least in Central Russia. The putative presence of its close relative, *S. sarrachoides* from South America, which is a naturalized alien in some parts of Europe, is also discussed. At present, the remaining species are considered casual aliens. *Solanum scabrum* has escaped from cultivation, whereas the other species have arrived with contaminated grain imported from North America.

## Introduction

*Solanum* L., with approximately 1,245 species widely distributed across the world, is the largest and most morphologically diverse genus of the Solanaceae (Hilgenhof et al. 2023). Besides economically important and globally cultivated crops, e.g., *S. lycopersicum* L., *S. melongena* L., and *S. tuberosum* L., some American species are noxious invasive weeds in the tropics and subtropics of the Old World, e.g., *S. elaeagnifolium* Cav. (Sheppard et al. 2006), *S. rostratum* Dunal (Datiles 2014; Ozuzu et al. 2024), and *S. viarum* Dunal (Waheed et al. 2025). In contrast, in temperate Europe, neophyte *Solanum* species are not considered invasive weeds (Lesieur et al. 2023), although some of them have a high potential to spread in various anthropogenic habitats (Edmonds and Chweya 1997; Weber and Gut 2005).

The latest taxonomic treatment of *Solanum* (excl. *Lycopersicon* Mill.) for Eastern Europe (Poyarkova 1981) counted 17 species, including five species from *S.* sect. *Dulcamara* (Dunal) Bitter, *S. melongena* L. from *S.* sect. *Melongena* (Mill.) Bitter, *S. tuberosum* L. from *S.* sect. *Tuberarium* (Dunal) Bitter, *S. sisymbriifolium* Lam. from *S.* sect. *Protocryptocarpum* Bitter, *S. cornutum* Lam. and *S. heterodoxum* Dunal from *S.* sect. *Androceras* (Nutt.) Bitter, and seven species from *S.* sect. *Solanum* sensu Poyarkova (1981). Two species, *S. melongena* and *S. tuberosum*, are widely cultivated crops. All five former species from *S.* sect. *Dulcamara* (Moench) Dumort. (sensu Poyarkova 1981), now included in the ‘Dulcamaroid clade’ (Bohs 2005), are currently treated as synonyms of *S. dulcamara* L. (Knapp 2013), native to Europe and temperate Asia. Three species from sect. *Solanum*, *S. luteum* Mill., *S. alatum* Moench, and *S. zelenetzkii* Pojark., are now considered synonyms of *S. villosum* Mill.; two others (*S. schultesii* Opiz, *S. humile* Bernh.) were merged with *S. nigrum* L. (Särkinen et al. 2018). Therefore, in the current circumscription, Poyarkova (1981) counted only four species that are alien to the European part of the former USSR, namely *S. heterodoxum*, *S. cornutum* (= *S. rostratum* Dunal), *S. luteum* (= *S. villosum*), and *S. sisymbriifolium*, and three of them, except *S. luteum*, are prickly species (belonging to subgen. Leptostemonum).

The current phylogeny-based classification of *Solanum* places the species of our study in two large clades. The large ‘Morelloid’ clade (Särkinen et al. 2018) comprises ~ 75 non-spiny, herbaceous or suffruticose species with simple or glandular, rarely branched hairs and mostly internodal inflorescences. It includes the species from section *Solanum*, related to *S. nigrum* and *S. villosum*. The species of this clade are distributed all over the world. Species of *S.* subg. Leptostemonum (Dunal) Bitter (incl. *S.* sect. *Androceras* and *Protocryptocarpum*) belong to the ‘Leptostemonum clade’, with the highest species richness in the Americas (Aubriot and Knapp 2022). This clade is the most species-rich group of the genus, comprising almost half of its currently recognized species (Gagnon et al. 2022). Morphologically, they are characterized by the presence of prickles, stellate or lepidote indumentum, and tapered anthers.

The first collections of new alien non-prickly *Solanum* species in our study area were made in the late 1980s and early 1990s, and these data were summarized several years later for North-West Russia (Tzvelev 2000), Central Russia (Mayorov 2014), and the North Caucasus (Ivanov 2019).

Nevertheless, the number of alien *Solanum* species in this territory is currently underestimated, and the taxonomic classification of some alien species remains unresolved (Table 1). This issue is further complicated by the fact that the latest revision of the Morelloid clade of *Solanum* (Särkinen et al. 2018), which contains many non-prickly species, does not include herbarium collections from Russia.

**Table 1.** Alien *Solanum* species recorded in the latest major checklists in European Russia and the North Caucasus.

Species	Central Russia (Mayorov 2014)	North-West Russia (Tzvelev 2000)	North Caucasus (Ivanov 2019)	present treatment
*Solanum cornutum* Lam.	absent	absent	present	misidentification for *S. rostratum* Dunal
*Solanum elaeagnifolium* Cav.	absent	absent	absent	accepted
*Solanum emulans* Raf.	absent	absent	absent	accepted
*Solanum heterodoxum* Dunal	absent	absent	absent	accepted
*Solanum judaicum* Besser	present	absent	absent	synonym of *S. nigrum* L.
*Solanum luteum* Mill.	present	present	present	synonym of *S. villosum* Mill.
*Solanum nigrum* L.	present	present	present	accepted
*Solanum nitidibaccatum* Bitter	absent	absent	absent	accepted
*Solanum physalifolium* Rusby	present	absent	absent	misidentification for *S. nitidibaccatum* Bitter
*Solanum rostratum* Dunal	present	present	present	accepted
*Solanum scabrum* Mill.	absent	absent	absent	accepted
*Solanum schultesii* Opiz	present	present	absent	synonym of *S. nigrum* L.
*Solanum sisymbriifolium* Lam.	absent	absent	present	accepted
*Solanum triflorum* Nutt.	present	present	present	accepted
*Solanum villosum* Mill.	present	present	present	accepted

The present study began in 2023 after the first author (APS) collected unusual *Solanum* specimens in Moscow and Saratov Oblasts (European Russia). Some material, including the collections of APS and specimens kept in LE and MW, was later brought to Sandra Knapp, a recognized expert in the Solanaceae, who provided an extensive revision of the *Solanum* specimens collected in European Russia and the North Caucasus. It turned out that many of these specimens were erroneously identified, and incorrect information was consequently used in checklists and floristic reports from some regions.

The ongoing confusion in the identification of *Solanum* specimens in the major Russian herbaria, the lack of Russian specimens in the latest revision of the Morelloid representatives of the genus, as well as the continuous emergence of new collections of alien species in European Russia and the North Caucasus prompted us to carry out a revision of the alien *Solanum* species occurring in this region. Besides the distributional data, we provide updated taxonomic and nomenclatural information, which is currently lacking in East European publications.

## Materials and methods

During the preparation of the present revision, the first author examined the material kept at BM, K, MHA, MOSM, MOSP, MW, MWG, LE, LECB, TK, and WIR and checked images sent from H, ORIS, PALE, PTZ, RV, RWBG, TUR, and UDU. Sandra Knapp, a Solanaceae expert, revised part of the material brought by APS to the NHM London. All new material collected by the authors is deposited at K, LE, PALE, and MW. Specimen information, complemented by documented observations available on citizen-science platforms (Plantarium: https://www.plantarium.ru/; iNaturalist: https://www.inaturalist.org/), is compiled in a dataset of Darwin Core-structured species occurrence records and observations (Sukhorukov 2025). No specimens are cited in the text.

This work is based exclusively on specimens and observations collected in the wild. Indoor-cultivated plants or major crop species occasionally found as escapees from cultivation in ruderal places (*S. melongena*, *S. lycopersicum*, *S. pseudocapsicum* L., and *S. tuberosum*) are excluded from our revision. Särkinen et al. (2018) circumscribed and mapped distributions for all accepted species of the ‘Morelloid clade’; however, these authors did not distinguish between native and secondary distribution areas for ‘old’ common weeds. Sennikov and Lazkov (2022) considered *S. nigrum* an archeophyte rather than a native species in Northern Eurasia. This species seems to be native to the Mediterranean basin and southern Asia (Särkinen et al. 2018); we consider it a putative archeophyte in the central and southern parts of Eastern Europe and the North Caucasus and a neophyte in the northern part of Eastern Europe. Records of this species are not cited or mapped in the present work due to its frequent weedy and ruderal occurrence in almost all parts of our study area, except the Arctic and Subarctic zones (Murmansk Oblast and Nenets Autonomous District; Poyarkova 1981). In the current work, we focus on neophytes and therefore omit *S. nigrum* from consideration. In European Russia and the North Caucasus, all species under consideration are alien (casual or naturalized). Since the taxonomic treatment includes only neophyte aliens, *S. nigrum* (archeophyte) is added to the identification key for comparison purposes only, because some related taxa are morphologically similar to this common species.

The alien species in this work are treated according to two major groups corresponding to their recent phylogenetic positions (Levin et al. 2006; Weese and Bohs 2007; Gagnon et al. 2022). Since this work serves as a taxonomic update to standard treatments in Eastern Europe, taxonomic synonyms used in this territory and their nomenclature (including types) are provided. For other synonyms, see Särkinen et al. (2018) and Knapp et al. (2019, 2023). The nomenclature is verified and updated when necessary.

The study area, the European part of Russia and the Russian North Caucasus, was selected for the following reasons: (1) Both large contiguous regions require a substantial taxonomic revision of alien *Solanum* species, since all previous treatments contained scarce or erroneous information on their taxonomic identity and distribution; (2) some species treated in this study have not been previously reported from either region. Regions outside the study area are also discussed to provide additional information on the distribution and invasiveness of some species.

Distribution maps are prepared using the SimpleMappr online tool (http://www.simplemappr.net). For some records, special symbols (squares or yellow dots) are used on maps. Squares denote field observations and images seen for *S. scabrum* Mill. in the Lower Volga Region, which were published without precise locality information in Sagalaev and Sukhorukov (2025), as well as literature references for *S. rostratum* in St. Petersburg (Tzvelev 2000) and the Kabardino-Balkar Republic (Chadaeva et al. 2019). A single yellow dot (in the case of *S. sisymbriifolium*) denotes an old record from the late 19^th^ century that has not been confirmed by recent observations.

The status of alien plant species was determined following the definitions proposed by Richardson et al. (2000). Published and personal observations were used to reconstruct the history and pathways of introduction of alien plant species into the study area. Pathways were coded according to Hulme et al. (2008) and interpreted as recommended by Harrower et al. (2018).

## Results

### Taxonomic treatment

Key to the major clades of alien *Solanum* species in European Russia and the North Caucasus

**Table:** 

1	Plants without prickles and stellate indumentum	***‘Morelloid clade’***
–	Plants with prickles; indumentum stellate or lepidote (in the latter case, trichome rays are fused at the base)	***‘Leptostemonum clade’***

### The ‘*Morelloid clade*’

#### Key to the alien *Solanum* species (‘*Morelloid clade*’) in European Russia and the North Caucasus

**Table:** 

1	Leaves pinnatifid; inflorescence with 1–3 flowers	***S. triflorum***
–	Leaves entire or lobed; inflorescence usually with more numerous flowers	**2**
2	Inflorescences with 8–12 or even more flowers; fruits 10–20 mm in diameter	***S. scabrum***
–	Inflorescences with (1–2)3–8 flowers; fruits 4–8 mm in diameter	**3**
3	Corolla white or light purple; fruits broadly ovoid or globose, yellow, or orange	***S. villosum***
–	Corolla white; fruits globose, black, green, or olive green	**4**
4	Corolla 9–13 mm in diameter; anthers 2–2.8 mm long	***S. nigrum***
–	Corolla 5–10 mm in diameter; anthers 1.0–1.8 mm long	**5**
5	Stem and leaves upright, almost glabrous or with scattered simple trichomes; calyx not accrescent; fruit black	***S. emulans***
–	Plants prostrate or ascending, somewhat sticky; leaves and stem indumentum of simple and glandular trichomes; calyx ± accrescent; fruit green or olivaceous	***S. nitidibaccatum***

##### 1. Solanum emulans

Taxon classificationPlantaeSolanalesSolanaceae

Raf., Autik. Bot.: 107 (1840).

5CDFDD02-58C7-5842-9E75-9627A914F2FC

Fig. 1

= Solanum nigrum var. virginicum L., Sp. Pl. 186 (1753) ≡ Solanum virginicum (L.) Hayne, Getreue Darstell. Gew. 2: ad tab. 40 (1809), non Solanum virginianum L. (1753) ≡ Solanum dillenianum Polg., Acta Horti Gothob. 13: 281 (1939). Type. [icon] “Solanum nigrum vulgari simile, caulibus exasperates,” cultivated in England, at James Sherard’s garden in Eltham (lectotype, designated by Edmonds in Jarvis (2007: 861): Dillenius, Hortus Elthamensis 2: 368, tab. 275, f. 356 (1732)).

###### Type.

USA. “Amer. bor.”, *C.S. Rafinesque s.n*. (neotype, designated by Knapp et al. (2019: 60): W 0009388).

**Figure 1.**
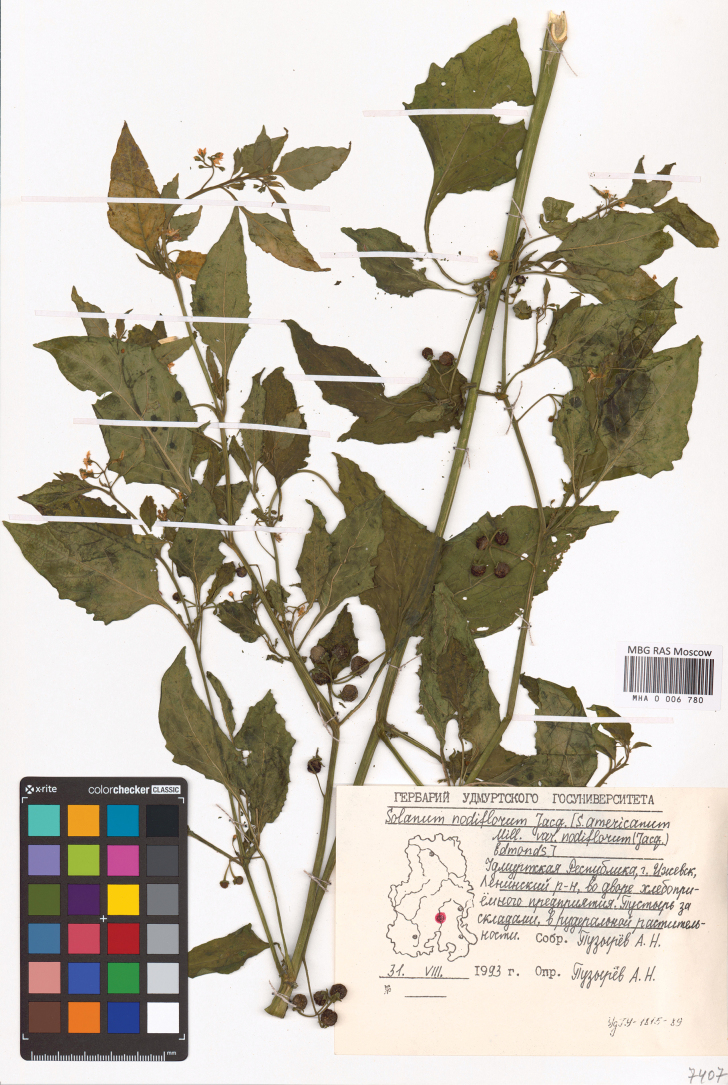
Herbarium specimen of *S. emulans* (MHA).

###### Description.

Upright non-sticky annuals up to 1 m tall; trichomes simple, scattered; leaves green, base broadly cuneate, margin entire or shallowly sinuate; each cyme with 3–6 flowers; corolla actinomorphic, white, 8.0–10.0 mm in diameter, anthers 1.0–1.5 mm long, equal; calyx not accrescent, much shorter than fruit, appressed to spreading; fruit 6–8 mm in diameter, black, rather opaque; mature fruits dropping with the pedicel, with 6–10 sclerotic granules.

###### Taxonomic note.

*Solanum emulans* can be distinguished from *S. nigrum* by the presence of sclerotic granules in berries and by very short anthers, 1.5 mm long or less (vs. over 2 mm long) (Knapp et al. 2019).

*Solanum americanum* differs from *S. emulans* in mature berries dropping without the pedicel, sclerotic granules absent or at most 2(4–6) (vs. dropping with the pedicel, sclerotic granules more than four, usually eight per berry), and calyx lobes strongly reflexed in fruit (vs. calyx lobes appressed to spreading in fruit) (Knapp et al. 2019). The leaves of *S. americanum* are rather attenuated toward the apex, usually with indistinct dentation, whereas those of *S. emulans* have a rather broadly triangular apex and prominent dentation.

Knapp et al. (2019) added *S. adventitium* Polg. to the synonymy of *S. emulans*. Polgár (1926) noted that *S. adventitium* is characterized by nearly entire to sinuate-dentate leaves and constantly six sclerotic granules in the fruit. On the basis of these characters, Edmonds (1972) referred this taxon to *S. americanum*, which also has subentire leaves and sometimes sclerotic granules in the fruit. Polgár (1926) inferred the origin (source of introduction) of *S. adventitium* as South America (Argentina), which is at odds with the distribution of *S. emulans*.

###### Nomenclatural note.

The earliest binomial for this species appears to be *Solanum virginicum* (L.) Hayne, which cannot be used because of the confusing similarity with its earlier near-homonym, *S. virginianum* L. This confusion can be seen in a number of old references, including Linnaeus (1771) and Rafinesque (1840).

###### Distribution.

A species native to North America (Knapp et al. 2019) and recently introduced in Europe, with very few scattered records known (Karlsson 1998; Verloove 2006; Puzyryov 2021b).

**Figure 2.**
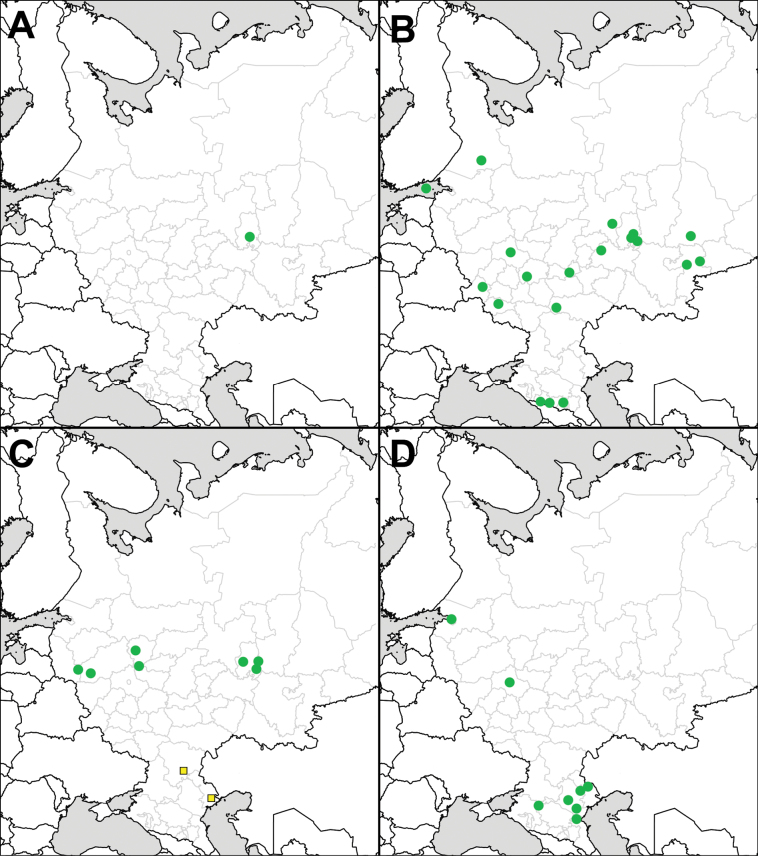
Records of *Solanum* species in European Russia: **A**. *Solanum emulans*; **B**. *S. nitidibaccatum*; **C**. *S. scabrum*; **D**. *S. triflorum*. Squares denote field observations and images seen for *S. scabrum* in the Lower Volga Region.

In European Russia (Fig. 2A), this species is known only from Izhevsk Town, Udmurt Republic, where it was introduced with imported grain, presumably from North America, as suggested by its appearance during the Soviet grain crisis (Puzyryov 2021b). The specimens were collected in the early 1990s but identified only recently, when a comprehensive taxonomic revision of the Morelloid clade became available (Särkinen et al. 2018). Puzyryov (2021b) identified his herbarium specimens on the basis of identification keys provided by Särkinen et al. (2018) and Knapp et al. (2019), owing to the presence of numerous sclerotic granules in the berries and prominently dentate leaves.

###### Ecology.

Ruderal places around grain storage and processing facilities and cargo railway stations.

###### Residence status.

Casual alien. The species can be evaluated as an ephemerophyte in the temperate climate.

###### Pathways of introduction.

Transport – Contaminant: seed contaminant.

Due to the records at grain processing facilities (Puzyryov 2021b), the species is a typical grain immigrant.

##### 2. Solanum nitidibaccatum

Taxon classificationPlantaeSolanalesSolanaceae

Bitter, Repert. Spec. Nov. Regni Veg. 11: 208 (1912) nom. cons.

3E66F9AE-27E1-52A4-9D68-214FAE92AB5A

Fig. 3

≡ Solanum physalifolium var. nitidibaccatum (Bitter) Edmonds, Bot. J. Linn. Soc. 92: 27 (1986).

###### Type.

Chile. [Without exact locality], 1829, *E.F. Poeppig s.n*. (lectotype, designated by Edmonds (1986: 27): W0004151; isolectotype: F [V0073346F, acc. # 875221]).

**Figure 3.**
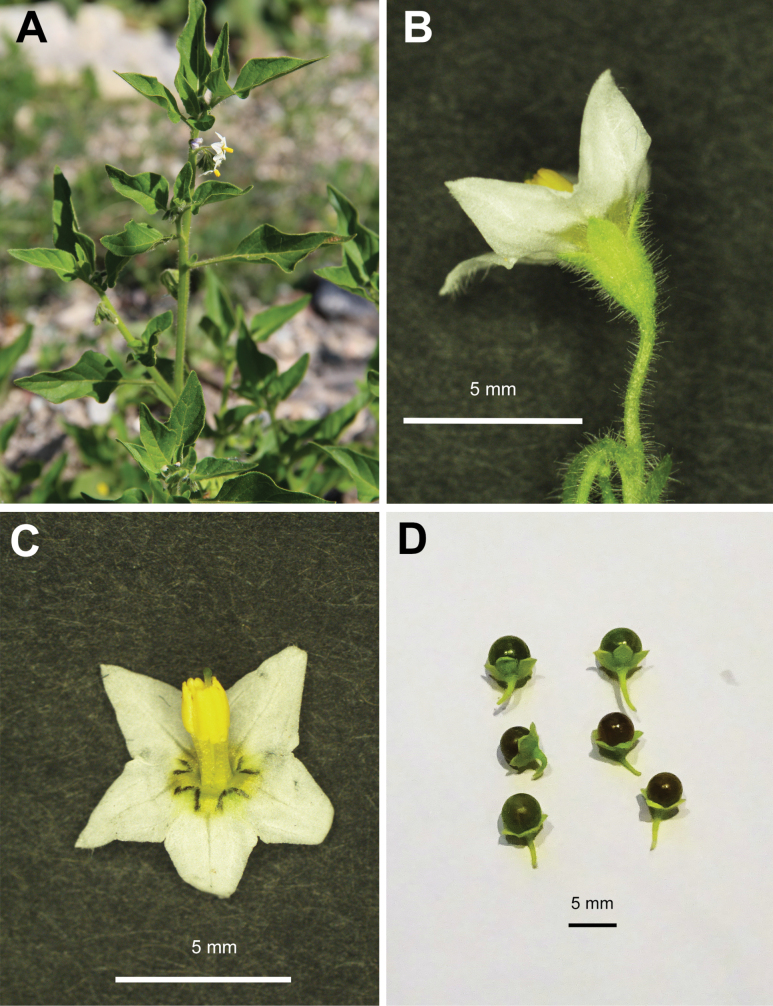
*Solanum nitidibaccatum*: **A**. upper part of the shoot with leaves and flowers; **B, C**. flowers; **D**. fruits. Photographers: D. Shilnikov (**A**; Terskol, Kabardino-Balkar Republic) and A. Sukhorukov (**B–D**; Moscow).

###### Description.

Upright, ascending or prostrate ± sticky annuals with stems up to 30 cm long; trichomes up to 2 mm long, spreading, simple and glandular especially abundant in upper stem parts and inflorescence; leaves light or bright green, concolorous, base broadly cuneate or truncate, margin entire or shallowly sinuate, pubescent; each cyme of 3–6(8) flowers; corolla actinomorphic, white, 5–9 mm in diameter, anthers 1.2–1.8 mm long, equal; calyx accrescent and not fully enveloping the fruit (up to 2/3 of fruit length), appressed but spreading in ripe fruits; fruit 5–8 mm in diameter, dark green or olivaceous, rather shiny when mature, sclereidal concretions present, 1–3.

###### Taxonomic note.

This South American species has been reported under the erroneous name *S. physalifolium* (e.g., Melnikov 2011; Sobrino Vesperinas and Sanz Elorza 2012; Galasso et al. 2018a; Mayorov 2018; Mayorov et al. 2019, 2020; Ebel et al. 2022) or accepted at a varietal rank as *S. physalifolium* var. nitidibaccatum (Karlsson 1998; Mito and Uesugo 2004; Verloove 2006).

According to current knowledge, both species are clearly distinct: *S. physalifolium* has anthers ca. 2 mm long (vs. less than 2 mm long in *S. nitidibaccatum*), and fruiting calyx lobes spreading, with very marked venation (vs. not markedly spreading lobes with indistinct venation) (Knapp et al. 2020). *Solanum physalifolium* is considered endemic to the Central and Southern Andes and is not found outside its native range (Särkinen et al. 2018; Galasso et al. 2018b, 2024).

Another South American species, *S. sarrachoides* Sendn., is morphologically very close to *S. nitidibaccatum* but differs from the latter by the calyx fully enveloping the fruit. Edmonds (1986) provided an extensive morphological analysis of both *S. nitidibaccatum* (as *S. physalifolium* var. nitidibaccatum) and *S. sarrachoides* and concluded that they cannot be merged into one variable species. Originating from South America, the latter species is a neophyte in some regions of the world, found in Europe in the early 20^th^ century (Särkinen et al. 2018), with further records in the late 20^th^ century, e.g., in southern Europe (Laorga 1983; Giráldez 1986) and Australia (Lepschi 1996). For a long time, this species was treated in a broad sense, including *S. nitidibaccatum* (e.g., Edmonds 1972; see also the discussion in Särkinen et al. 2018). For this reason, some records of this species outside South America may belong to *S. nitidibaccatum*. Interestingly, *S. nitidibaccatum* and *S. sarrachoides* were first discovered in Europe approximately at the same time (according to the specimens cited in Särkinen et al. 2018), in the late 19^th^ (*S. nitidibaccatum*) and early 20^th^ centuries (*S. sarrachoides*). Specimens with an accrescent calyx fully enveloping or even exceeding the fruits were seen by us, e.g., from Sweden (Blom 1936, K001150943) and the United Kingdom (Melville 1927, K001150934, K001150935; Hubbard et al. 1929, K001150930; Sandwith and Milne-Redhead 1945, K001150936–K001150938; Lousley 1954, K001150933; Dony 1955, K001150940). So far, no records of *S. sarrachoides* are available from Eastern Europe.

*Solanum nitidibaccatum* is characterized by vigorous growth and an unusual branching pattern, which readily distinguishes it from the similar and common black-fruited weed *S. nigrum* (Bosek 1989). When distinguished on the basis of vegetative characters, *S. nitidibaccatum* was sometimes misidentified as *S. villosum* in floristic explorations, e.g., Sapronova and Safonov (2000), Poluyanov (2005), and Kravchenko (2024).

###### Distribution.

Native to temperate South America; naturalized alien in Europe, North America, Australia, and New Zealand.

*Solanum nitidibaccatum* was unintentionally introduced to Germany at the end of the 19^th^ century (Militzer 1964). In the early 20^th^ century, it was also collected in several European countries, including Sweden, France, Germany, the Netherlands, and the United Kingdom (Hegi 1927; Särkinen et al. 2018). The majority of its collections in Europe date from the 1990s (Särkinen et al. 2018).

Outside Europe, *S. nitidibaccatum* is widely distributed as an alien species in many parts of North America, temperate and subtropical Asia, and Australia (Mito and Uesugo 2004, as *S. physalifolium* var. nitidibaccatum; Särkinen and Knapp 2022; Amin et al. 2025). It has also recently been discovered in North Africa (Morocco: Khamar et al. 2024).

In Eastern Europe, the species was reported from Belarus (Dubovik et al. 2014) and Moldova (Puzyryov 2021a and references therein). In European Russia (Fig. 2B), *S. nitidibaccatum* has been found in Bryansk Oblast (Bosek 1989), Chelyabinsk Oblast (Ebel et al. 2022, as *S. physalifolium*), Kirov Oblast (new record), Republic of Karelia (Kravchenko 2024, as *S. villosum*), Kursk Oblast (Sapronova and Safonov 2000, as *S. villosum*; Mayorov 2018, as *S. physalifolium*), Republic of Mordovia (Silaeva et al. 2016, as *S. physalifolium*), Moscow Oblast (Mayorov et al. 2012, 2019, 2020, as *S. physalifolium*), Leningrad Oblast (new record), Ryazan Oblast (Mayorov 2018, as *S. physalifolium*), Saratov Oblast (Puzyryov 2021a), Sverdlovsk Oblast (new record), Republic of Tatarstan (new record), and Udmurt Republic (Ilminskikh et al. 1998; Puzyryov 2006, 2009, 2021a, 2025; Baranova and Puzyryov 2012). In the North Caucasus (Fig. 2B), *S. nitidibaccatum* was first recorded from the Karachay-Cherkess Republic (Puzyryov 2021a) and later from the Kabardino-Balkarian Republic and the Republic of North Ossetia–Alania (new records).

In Russia, *Solanum nitidibaccatum* was first discovered in Bryansk Oblast in 1987 (Bosek 1989) and shortly thereafter in many other administrative units. It is largely found in forest-steppe and steppe zones of European Russia but also occasionally occurs farther north, up to Karelia. In the North Caucasus, the species is found in foothills and uplands with adequate precipitation.

Perhaps the most abundant observations of *S. nitidibaccatum* originate from the Udmurt Republic, where the species has been repeatedly found, often in large quantities, on cultivated and waste lands, in ruderal places, and along roadsides (reviewed by Puzyryov 2021a).

In Leningrad Oblast, this species was first found only in 2014–2015 on Gogland Island in the eastern Gulf of Finland (new record). Its previous report from this administrative territory (Saarisalo-Taubert 1967) is erroneous. That record was based on a specimen (Viipuri [now Vyborg town], 1907, *L. Oesch s.n*. TUR – image!) corresponding to *S. nigrum*, more densely covered with trichomes and with clearly longer anthers (at least 2 mm long).

A putative first observation of *S. nitidibaccatum* near Moscow (Lytkarino township) was made by M. Nuraliev, and a photograph documenting this record was reproduced by Mayorov et al. (2020, as *S. physalifolium*). The voucher for another record [Moscow City, Verkhnyaya Khokhlovka St., lawn, one individual, 18 August 2015, *V. Bochkin*, MHA] cited by Mayorov et al. (2019) has not been found in the herbarium. Our record is therefore the first specimen-documented record of *S. nitidibaccatum* for Moscow City and Moscow Oblast as a whole.

Puzyryov (2021a) speculated that *S. nitidibaccatum* may already have occurred in territories adjacent to the Udmurt Republic, e.g., the Republic of Tatarstan, Kirov Oblast, and Sverdlovsk Oblast. We confirm the occurrence of the species in these territories on the basis of documented observations available on iNaturalist.

###### Ecology.

Waste and disturbed ground, cultivated land (fields, commercial and kitchen gardens, flower gardens), roadsides, lawns, and spreading to disturbed natural grasslands.

###### Residence status.

The residence status of this species is variable throughout Europe. In Germany, it seems to have naturalized by the mid-20^th^ century (Militzer 1964). The same status has been recently reported from Belgium (Verloove 2006, as *S. physalifolium*) and Italy (Galasso et al. 2018a, as *S. physalifolium*; Galasso et al. 2024), but the species appears to be a rare casual alien in Albania (Barina et al. 2014, as *S. physalifolium*).

Collections from numerous regions may confirm the species’ naturalization in European Russia and the North Caucasus, especially in the Udmurt Republic, where extensive weedy populations have been found (Melnikov 2011, as *S. physalifolium*; Puzyryov 2021a). Similar observations were obtained from the South Ural and South Siberia, where the species spreads actively (Ebel et al. 2022, with references therein; Chepinoga et al. 2024). On the other hand, the species is recorded as clearly casual (present as single individuals along roadsides and on waste ground) in other regions, e.g., the Republic of Karelia, the Moscow Region, and the Republic of Tatarstan.

###### Pathways of introduction.

Transport – Contaminant: seed contaminant.

Puzyryov (2021a) inferred from the species’ occurrence in cornfields that it likely originated as a grain contaminant of North American origin in the Udmurt Republic (in the 1990s, Russia actively imported grain from North America). This appears to be its major introduction pathway in Russia. Nevertheless, many recent records from various areas were made in ruderal habitats, e.g., roadsides, lawns, and disturbed grassy landscapes, but its presence in cultivated areas, especially in potato fields (Vershinin and Kuzmin in Nobis et al. 2020), is also notable.

Further local dispersal of the species is observed with agricultural transport and along roadsides (Puzyryov 2025).

##### 3. Solanum scabrum

Taxon classificationPlantaeSolanalesSolanaceae

Mill., Gard. Dict. ed. 8: Solanum no. 6 (1768).

9A69A7F3-3B31-5DAF-B4BD-C44BF4695FB9

Fig. 4

= Solanum melanocerasum All., Auct. Syn. Meth. Stirp. Hort. Regii Taur.: 664 (1774). Type. “Solanum guineense, fructo magno, instar cerasi nigerrimo, umbellato” cultivated in England in John Sherard’s garden in Eltham, Herb. Dillenius 336 (neotype, designated by Edmonds (2012: 129) as “lectotype”: OXF [Dill. HE-274-234]).

###### Type.

Cultivated at Chelsea Physic Garden, Herb. Miller (lectotype, designated by Henderson (1974: 61) as “type”: BM000847083).

**Figure 4.**
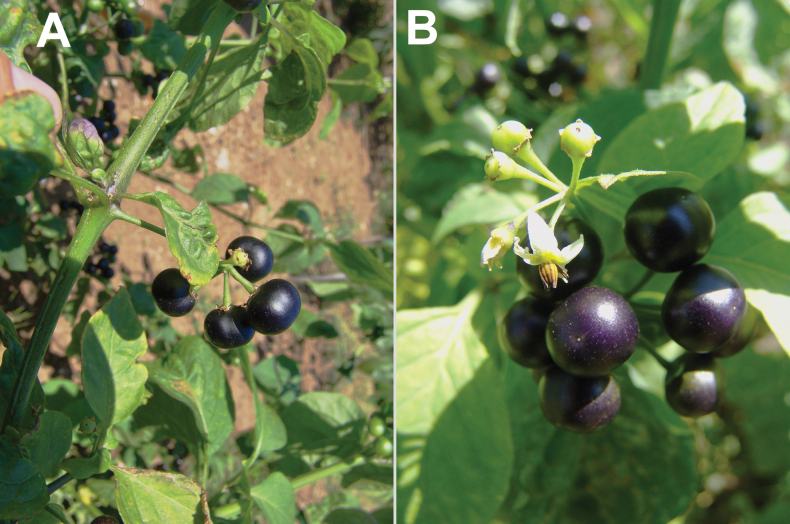
*Solanum scabrum*: **A**. leaves and fruits; **B**. fruits. Photographer: V.A. Sagalaev (Volgograd Oblast).

###### Description.

Upright and robust, non-sticky annuals or (in warmer regions) short-lived perennials up to 150 cm tall; trichomes simple; leaves green or somewhat purple, ± concolorous, base truncate, margin entire or rarely shallowly sinuate, glabrous or with short trichomes located mainly along the veins; each cyme of 8–12 or even more flowers (in cultivated varieties); corolla actinomorphic, white or occasionally lilac, 7–12 mm in diameter, anthers 2–3 mm long, equal; calyx not accrescent, not enveloping ripe fruits (calyx segments spreading or slightly reflexed); fruit 10–20 mm in diameter, purple-black, ± glossy when mature, sclereidal concretions absent.

###### Taxonomic note.

*Solanum scabrum* has been known and reported under a number of synonyms, such as *S. melanocerasum* (Baranova and Puzyryov 2012). Puzyryov (2021b) noted that it was confused with *S. retroflexum* in cultivation, but the latter can be readily distinguished by its shorter anthers (1.3–1.8(–2.0) mm long vs. 2–3 mm long in *S. scabrum*) (Särkinen et al. 2018).

###### Distribution.

This species is native to tropical Africa (Särkinen et al. 2018), where it is widely cultivated for its edible leaves as well as for other purposes (Manoko et al. 2008, with references therein). It is also cultivated in other regions, e.g., Australia (Symon 1981) and Great Britain (Stace 2010), but has not yet naturalized there. It is sometimes cultivated in Russia as an ornamental or edible plant under the name “Sun Berry” or “Canadian Blueberry” (Puzyryov 2021b; Tremasova 2023).

Native to Tropical Africa; alien in Europe, North America, and Australia.

In European Russia, the species was first reported from the Udmurt Republic (Baranova and Puzyryov 2012, as *S. melanocerasum*). Currently, it is also known from Astrakhan Oblast (Sagalaev and Sukhorukov 2025), Tver Oblast (Notov 2009; Mayorov 2018, both as *S. americanum*), the Udmurt Republic (Puzyryov 2021b), Volgograd Oblast (Sagalaev and Sukhorukov 2025), and Yaroslavl Oblast (Tremasova 2023).

###### Ecology.

Landfills, cultivated land. Because of its cultivation, species occurrences are largely confined to landfills, where they originate from garden waste (Puzyryov 2021b; Tremasova 2023).

###### Residence status.

Casual alien. Based on its tropical origin, the species cannot survive in the temperate climate for a long time (Sagalaev and Sukhorukov 2025).

###### Pathways of introduction.

Escape from confinement: ornamental purpose other than horticulture; agriculture.

This species is an ergasiophyte, rarely found near cultivated areas in European Russia.

##### 4. Solanum triflorum

Taxon classificationPlantaeSolanalesSolanaceae

Nutt., Gen. N. Amer. Pl. 1: 128 (1818).

C194BDF3-2DD6-5EB2-AE14-0F9BD88C12E8

Fig. 5

###### Type.

USA. North Dakota, near Fort Mandan, [*Lewis & Clark s.n*.] (lectotype, designated by Hunziker (1989: 189); superfluously redesignated by Barboza et al. (2013: 260): PH00030496).

**Figure 5.**
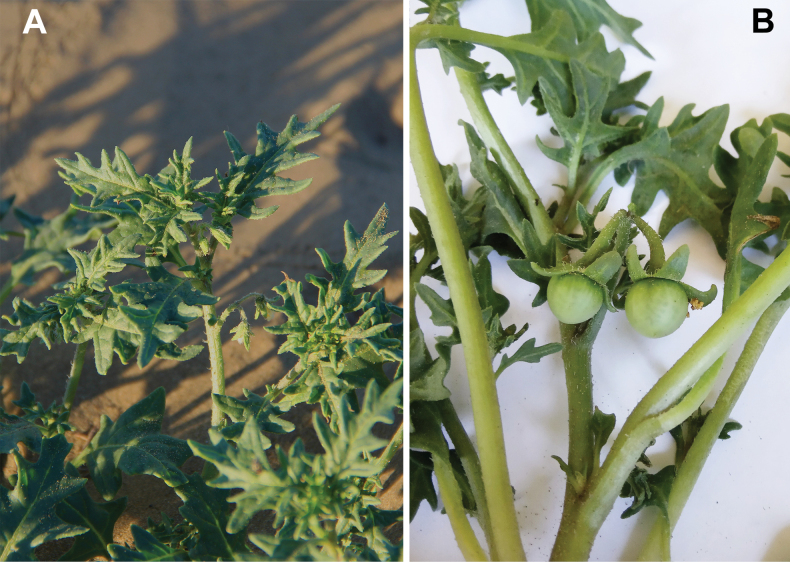
*Solanum triflorum*: **A**. upper part of the shoot with leaves and flowers; **B**. shoot with unripe fruits. Photographer: D. Shilnikov (Neftekumsk, Stavropol’ Krai).

###### Description.

Prostrate or ascending, non-sticky annuals up to 50 cm long; trichomes mostly simple, occasionally glandular; leaves green, concolourous, pinnatifid, base attenuate, lobes acutish, pubescent; each cyme of 1–3 flowers; corolla actinomorphic, white, 10–15 mm in diameter, anthers ± 3.0 mm long, equal; calyx slightly accrescent, not enveloping the fruit completely, spreading or reflexed in fruiting; fruit 9–14 mm in diameter, dark green, dull, sclereidal concretions abundant, 10–35.

###### Distribution.

Temperate and subtropical parts of the Americas; alien in Europe, South Siberia, Australia, and Africa.

This species has a disjunct native distribution in temperate and subtropical parts of North and South America and is often found in anthropogenically disturbed landscapes (Knapp et al. 2023). In Europe, *S. triflorum* was first introduced to Belgium in 1893 with goods and raw materials from the Americas, and the species has been evaluated as a naturalized alien in that country (Verloove 2006). To date, *S. triflorum* has been predominantly recorded in Western and Central Europe (Särkinen et al. 2018).

The first collections from Russia are known from 1943, from Omsk Oblast, South Siberia (seen in herb. TK!), where *S. triflorum* was introduced with crop seeds. In the 1960s, it was also collected close to South Siberia, in North-East Kazakhstan (Pavlodar Oblast, Bayanaul district, near Karazhar vill., in kitchen gardens, 28 August 1966, Shkuratenko, MWG, as *S. decipiens*), but not included in the Kazakhstan flora checklist (Abdulina 1998); this historical specimen represents a new country record for Kazakhstan. *Solanum triflorum* started to spread widely in South Siberia in the late 20^th^ century and became a naturalized alien species in the Altai Republic, the Republic of Buryatia, and Irkutsk Oblast (Teryokhina 2016; Plikina and Efremov 2017; Kolesova et al. 2024).

In European Russia (Fig. 2D), the species was first collected in Moscow Oblast (Mayorov et al. 2012) and Leningrad Oblast (Tzvelev 2000), but recent findings (after 1999) are known only from the southern part of Russia (the Republic of Kalmykia and Astrakhan Oblast: Ivanov 2001; Sagalaev et al. 2012), whereas only scattered records have been reported from the North Caucasus: Chechen Republic (Shkhagapsoev et al. 2022), Republic of Dagestan (new record), and Stavropol Krai (new record).

###### Ecology.

Sands, sandy steppes, waste ground, grain-processing facilities, and roadsides. Records in Moscow and Leningrad Oblasts were made near railway tracks, whereas records in the steppe and desert zones of southern Russia are confined to other habitats (roadsides, semi-natural landscapes, especially sands).

###### Residence status.

Casual alien in the forest zone of European Russia; more frequent, naturalized, and currently expanding in the Lower Volga Region and semi-deserts of the North Caucasus. We consider *S. triflorum* a casual xenophyte in the north but established in natural habitats and spreading in the Lower Volga Region and the North Caucasus.

###### Pathways of introduction.

Transport – Contaminant: seed contaminant.

Considering its occurrences near grain stations, railway stations, and along roads, the species can be considered a grain immigrant in the territory.

##### 5. Solanum villosum

Taxon classificationPlantaeSolanalesSolanaceae

(L.) Mill., Gard. Dict. ed. 8: Solanum no. 2 (1768).

776F399D-52F8-514A-BB0B-7FC73240CAC7

Fig. 6

≡ Solanum nigrum var. villosum L., Sp. Pl. 1: 186 (1753).
= Solanum luteum Mill., Gard. Dict. ed. 8: Solanum no. 3 (1768). Type. “Solanum officinarum; acinis luteis. C.B. 166,” numbered 691 and dated 1735, from the Worshipful Society of Apothecaries (lectotype, designated by Edmonds (1979: 214): BM000942567).
= Solanum alatum Moench, Methodus: 474 (1794), nom. cons. Type. Pakistan. Balochistan, Quetta, 30 km E Gumbaz, 1050 m, 30°02'N, 69°00'E, 17 May 1965, *K.H. Rechinger 29684* (conserved type: W acc. # 1972-0017910).
= Solanum humile Bernh. ex Willd., Enum. Pl.: 236 (1809), non Lam. (1794), nom. illeg. Type. Cultivated in Berlin Botanic Garden, from “Europa australi” [southern Europe], *Anonymous s.n.*. (lectotype, designated by Edmonds (2012: 132): B [B-W04367 sheet 2]; isolectotype: B [B-W04367 sheet 1]).
*= Solanum transcaucasicum* Pojark., Bot. Mater. Gerb. Bot. Inst. Komarova Akad. Nauk SSSR 17: 332 (1955). Type. Azerbaijan. “Talysh bei Tatuni [Tatoni, 38.65 N, 48.4 E]”, *R.F. Hohenacker s.n*. (holotype: LE).
*= Solanum zelenetzkii* Pojark., Bot. Mater. Gerb. Bot. Inst. Komarova Akad. Nauk SSSR 17: 336 (1955). Type. Crimea, Yalta, Oreanda, 21 August 1906, *K. Golde s.n*. (holotype: LE).
*= Solanum woronowii* Pojark., Bot. Mater. Gerb. Bot. Inst. Komarova Akad. Nauk SSSR 17: 337 (1955). Type. Transcaucasus, Abkhasia, opp. Gagry, in declivibus ad marginem viae, 30 October 1954, *A. Pojarkova 6* (holotype: LE).

###### Type.

[icon] “Solanum annuum hirsutus, baccis luteis,” cultivated in England, at James Sherard’s garden in Eltham (lectotype, designated by Henderson (1974: 56): Dillenius, Hortus Elthamensis 2: 366, tab. 274, fig. 353 (1732)).

**Figure 6.**
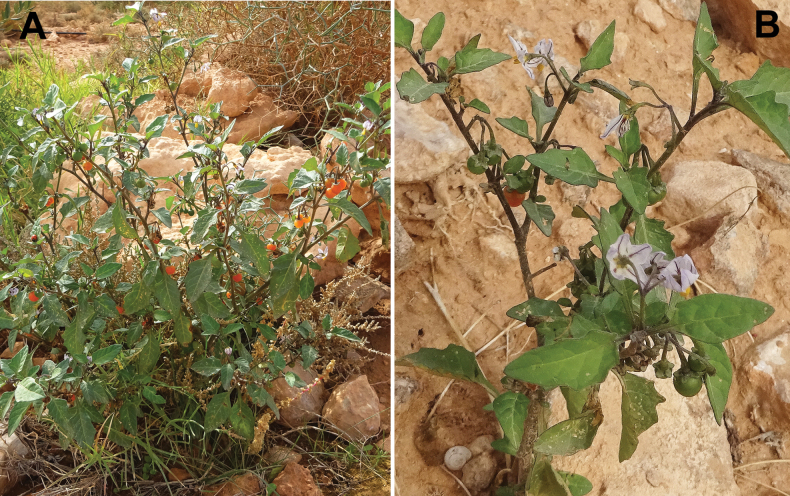
*Solanum villosum*: **A**. plant in flowering and fruiting; **B**. close-up of the leaves and flowers. Photographer: A. Sukhorukov (Lamrija, Guercif Province, Morocco).

###### Description.

Upright annuals up to 50 cm tall; trichomes mostly simple, short or long, occasionally glandular; leaves dull green, concoloured, sinuate-dentate or entire, ovate, base attenuate; each cyme of 2–5(6) flowers; corolla actinomorphic, white or light purple, 10–15 mm in diameter, anthers 1.8–2.2 mm long, equal; calyx not accrescent, small and not enveloping the fruit completely, spreading or reflexed in fruiting; fruit 6–8 mm across, broadly ovoid or roundish, orange or red, dull, sclereidal concretions usually absent.

###### Taxonomic note.

In its vegetative characteristics, *S. villosum* is very similar to *S. nigrum*, and the color and shape of ripe berries are the main differences between these species. Sometimes these species are mistaken for each other; remarkably, the only record of *S. villosum* from Leningrad Oblast (Viipuri [Vyborg] town, 1934, *V. Erkamo s.n*. (H766135)), which was published by Saarisalo-Taubert (1967), is rejected here because it appears to belong to a more hirsute variety of *S. nigrum*.

###### Nomenclatural note.

Särkinen et al. (2018) cautiously treated *S. villosum* Mill. and *S. nigrum* var. villosum L. as heterotypic synonyms because Miller’s protologue included no direct or indirect reference to, or mention of, the Linnaean variety. However, Miller (1768) noted that he adopted the Linnaean nomenclature, and his acceptance of the Linnaean taxon with the same final epithet may be treated as an indication of Miller’s potential intent to publish a new combination rather than a new species name in its own right (Art. 41.4 of the International Code of Nomenclature, ICN: Turland et al. 2025).

The lectotype of *S. luteum* is specimen no. 691, presented to the Royal Society in London by the Worshipful Society of Apothecaries in 1735 (Rand 1737), collected from a plant cultivated in England at the Chelsea Physic Garden, of American origin (Miller 1768), and identified according to Bauhin (1623: 166). The specimen was originally kept at the Society but transferred to the British Museum in 1781 (Stungo 1993).

###### Distribution.

This species is native to the Mediterranean Region, the Caucasus, and the Irano-Turanian Region, whereas it is probably alien in the North Caucasus, Central Asia, Africa, and India. Its alien occurrences are scattered in European Russia but are more common in the North Caucasus.

Records of this species in the Russian North Caucasus date back to the early 20^th^ century, but in Transcaucasia (Armenia, Georgia, and Azerbaijan) it was already noted by Bieberstein (1819, as *S. nigrum* var. villosum). The first collections available at LE date back to the middle of the 19^th^ century. Grossheim (1926) supposed that *S. villosum* (as *S. flavum* Kit.) is an eastern Mediterranean floristic element. Because of its absence from natural habitats, this species was evaluated as an alien species in Central Asia (Sennikov and Lazkov 2022, 2024) and in Belgium (Verloove 2006). In contrast, Särkinen et al. (2018) outlined the native range of *S. villosum* across West and Central Europe, Africa, Central Asia, and Hindustan.

**Figure 7.**
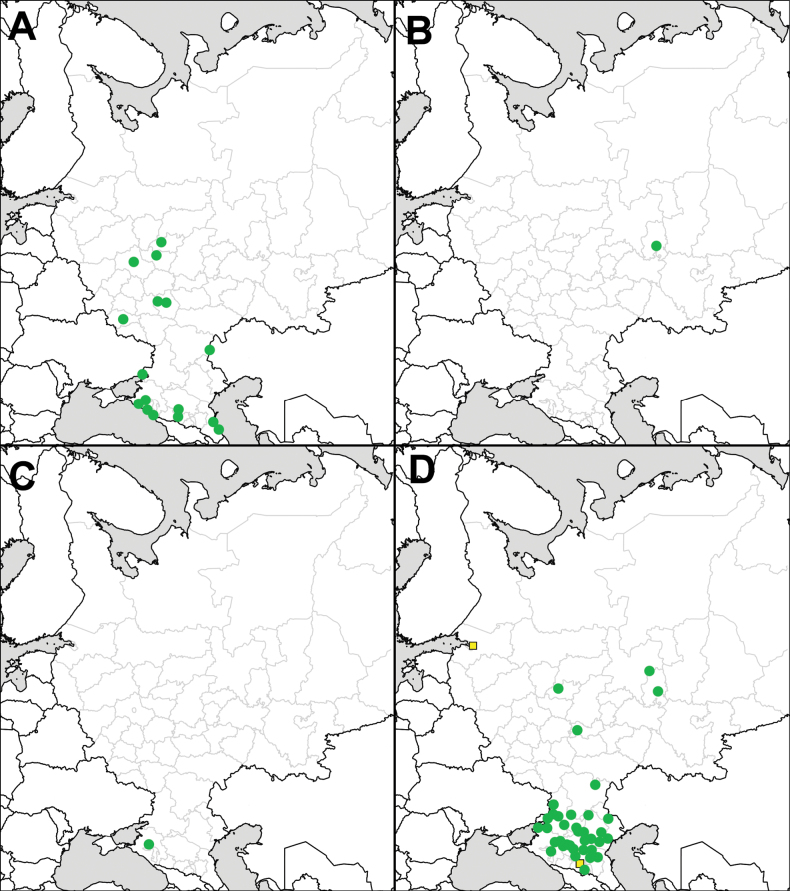
Records of *Solanum* species in European Russia: **A**. *Solanum villosum*; **B**. *S. elaeagnifolium*; **C**. *S. heterodoxum*; **D**. *S. rostratum*. Yellow squares denote data from references.

In European Russia (Fig. 7A), very scattered records of the species are known from Ivanovo Oblast (Borisova 2004, as *S. luteum*), Kursk Oblast (Zolotukhin 2013, as *S. humile*), Moscow Oblast (Mayorov et al. 2012, as *S. villosum*), Rostov Oblast (Poyarkova 1981, as *S. luteum*), Tambov Oblast (Sukhorukov 2010, as *S. judaicum* and *S. luteum*), Vladimir Oblast (Borisova and Senyushkina 2008, as *S. luteum*), and Volgograd Oblast (Poyarkova 1981, as *S. luteum*).

Kravchenko (2024) erroneously reported a casual occurrence of *S. villosum* from Petrozavodsk, Republic of Karelia. This specimen belongs to *S. nitidibaccatum*.

In Krasnodar Krai, the species is currently common in some areas, e.g., between Anapa town and Dzhubga village (D. Shilnikov, pers. obs.). In European Russia (Fig. 7A), it is clearly a neophyte, whose active spread started in the late 20^th^ century. Remarkably, by that time it was not known in the Lower Don area in the southern part of European Russia (Zozulin and Fedyaeva 1985).

###### Ecology.

Waste ground, roadsides, kitchen gardens.

###### Residence status.

The residence status of *S. villosum* in the southern parts of European Russia can be defined as that of a naturalized alien. It is clearly a neophyte in European Russia, where it has been found mostly since the early 2000s.

The species is probably an archeophyte in the North Caucasus.

###### Pathways of introduction.

Transport – Contaminant: seed contaminant.

The species was usually found on waste lands, landfills, and roadsides. This indicates its possible arrival as a seed contaminant, being a garden weed in the southern regions.

### The ‘*Leptostemonum clade*’

#### Key to the alien *Solanum* species (‘*Leptostemonum clade*’) in European Russia and the North Caucasus

**Table:** 

1	Rhizomatous subshrub; leaves entire, crenate, or sinuate; indumentum of lepidote and stellate trichomes	***S. elaeagnifolium***
–	Annual herbs; leaves lobate to pinnatisect or bipinnatisect; indumentum of stellate trichomes, sometimes intermixed with glandular and simple trichomes	**2**
2	Indumentum of stellate, glandular, and simple trichomes; leaf lobes slightly acute; corolla violet or white	**3**
–	Indumentum of stellate trichomes; leaf lobes blunt; corolla yellow	***S. rostratum***
3	Corolla radially symmetric; all anthers equal in length; fruits exserted from calyx at maturity	***S. sisymbriifolium***
–	Corolla bilaterally symmetric; one stamen longer than others, curved; fruits inserted in calyx at maturity	***S. heterodoxum***

##### 6. Solanum elaeagnifolium

Taxon classificationPlantaeSolanalesSolanaceae

Cav., Icon. 3: 22 (1795).

8C2F1FE8-10AB-5B9D-A497-22A9841C837C

Fig. 8

###### Type.

Cultivated in Madrid from “America calidiore” [“del viaje de los españoles alrededor del mundo, Cult. en el R. J. Bot. 1793”], *Anonymous s.n.*. (lectotype, designated by Knapp (2007: 198): MA-476348-2; isolectotype: MA-476348-1).

**Figure 8.**
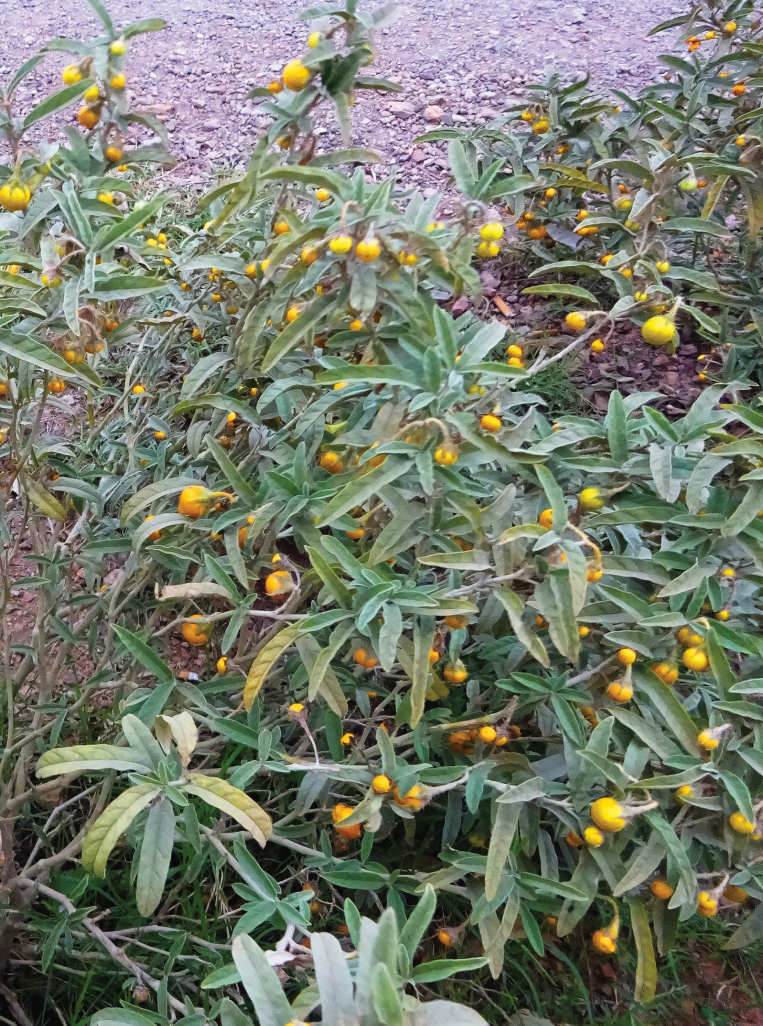
*Solanum elaeagnifolium*. Photographer: A. Sukhorukov (M’Hamid El Ghizlane, Morocco).

###### Description.

Rhizomatous subshrub up to 50 cm tall, ± prickly, especially in lower parts of the stem, covered with lepidote (peltate) and stellate hairs; leaves grey from both sides, oblong or elliptic, entire, crenate, or sinuate; each cyme of 4–6 flowers; corolla actinomorphic, usually violet or purple, rarely white, 25–35 mm in diameter; all anthers ± equal, 6–10 mm long; fruit 7–12 mm in diameter, exserted, leathery, orange with sticky pulp, dull, sclereidal concretions absent.

###### Distribution.

This species is native to South America and alien in many warm temperate and subtropical regions of the world.

The earliest European records date back to the late 18^th^ century in Spain and the mid-19^th^ century in France, but its spread to the Mediterranean area probably began in the mid-20^th^ century (Uludag et al. 2016; see also the discussion in Knapp et al. 2017). To date, *S. elaeagnifolium* is considered an invasive species inhabiting disturbed places across the Mediterranean area (e.g., Sforza and Jones 2007; Brunel 2011; Krigas et al. 2023). Recent documented records of the species close to Russia have been reported from Turkey (İlçim and Behçet 2007) and Azerbaijan (Zernov and Mirzayeva 2016). In Azerbaijan, *S. elaeagnifolium* has invaded semi-deserts with dominant native species such as *Caroxylon dendroides* (Pall.) Tzvelev, *Alhagi pseudalhagi* (M.Bieb.) Desv., and *Artemisia fragrans* Willd. (V. Karimov in herb. LE). The invasion of *S. elaeagnifolium* in Azerbaijan, as well as a recent observation in Georgia (iNaturalist: https://www.inaturalist.org/observations/179191722), makes further expansion into the plains of the Republic of Dagestan (Russia) predictable. To date, *S. elaeagnifolium* has been recorded in the North Caucasus (Chechen Republic; Shkhagapsoev et al. 2022), but we have not seen the relevant herbarium specimens.

Its only records in European Russia (Fig. 7B) are known from the Udmurt Republic, where the species was found in 1985 and 1989 in the territory of grain-processing facilities (Tuganaev and Puzyryov 1988; Baranova and Puzyryov 2012). These records are linked with the large-scale late Soviet import of North American grain, which was responsible for the casual introduction of several exotic alien plants and, apparently, the mass invasion of the noxious weed *Bidens frondosa* L. (Sennikov and Lazkov 2022).

###### Ecology.

Waste ground, roadsides, railroad beds, grain-processing facilities.

###### Residence status.

Casual alien in Central Russia. In the Udmurt Republic, *S. elaeagnifolium* is not able to survive in the temperate climate with cold winters.

###### Pathways of introduction.

Transport – Contaminant: seed contaminant.

The species is a typical grain immigrant in Russia.

##### 7. Solanum heterodoxum

Taxon classificationPlantaeSolanalesSolanaceae

Dunal, Hist. Nat. Solanum : 235 (1813).

26A53034-0EC2-5DB1-B770-B02C50B8BF1B

Fig. 9

###### Type.

[icon] *Solanum heterodoxum* (lectotype, designated by Whalen (1979: 413): Hist. Nat. Solanum, tab. 25 (1813).

**Figure 9.**
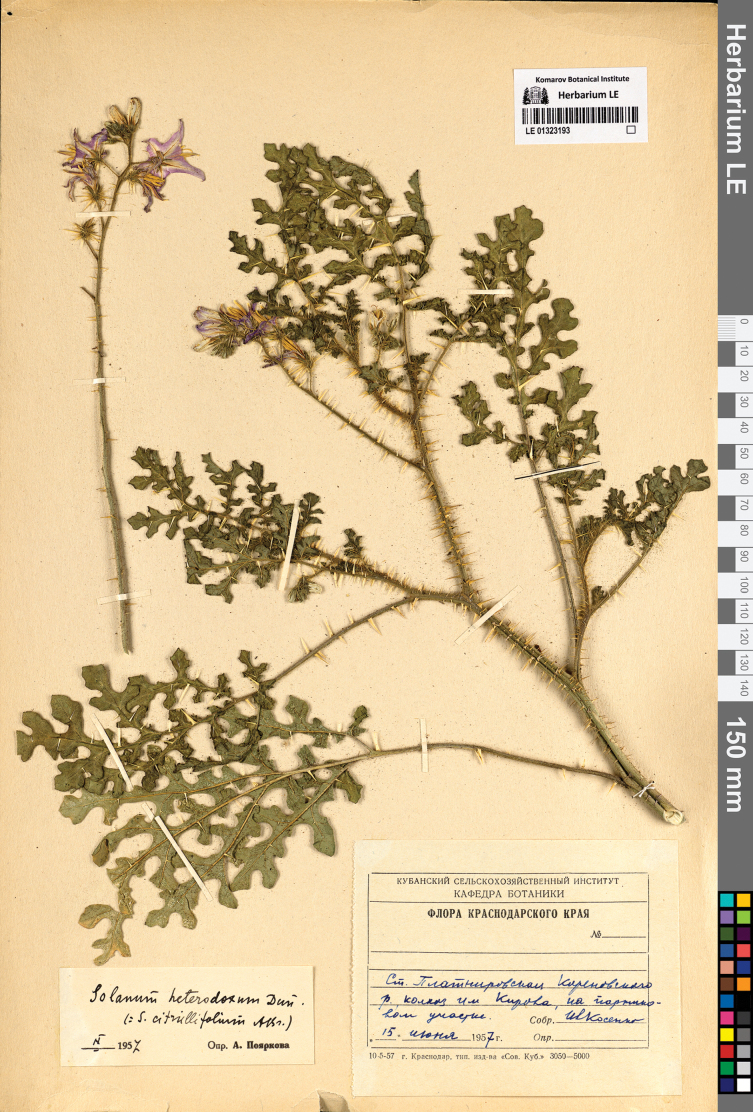
Herbarium specimen of *S. heterodoxum* (LE).

###### Description.

Prickly and sticky annual up to 70 cm tall; prickles basally slightly thickened; indumentum of glandular hairs mixed with stellate and simple hairs; leaves green, concolorous, pinnatisect or bipinnatisect, leaf lobes acutish; each cyme of 5–9 flowers; corolla violet, 10–17 mm in diameter, somewhat zygomorphic; one of five anthers longer, 3.5–5.0 mm long (all other anthers 2.5–4.0 mm long); fruit 10–13 mm in diameter, enclosed in a prickly calyx, dry, irregularly splitting at maturity, sclereidal concretions absent.

###### Distribution.

The native distribution area of the species is North America (Mexico and the southern states of the USA). It seems to be a rare alien in southern Europe (e.g., Poyarkova 1981; Galasso et al. 2018a, 2024; Stoyanov et al. 2021). A putative first collection of this species in Europe was made in Italy in 1860 (G. Galasso, pers. comm.) and subsequently in Crimea (Feodosiya town, seacoast, 17 July 1893, *O.A. Fedchenko & B.A. Fedchenko*, LE). In the early period, the species was also cultivated in Kiev Oblast from seeds collected in Podolia (Ukraine) in 1928, on a field with *Sorghum* sp. (LE).

In the North Caucasus (Fig. 7C), only a single record is known. In 1957, the species was found in Krasnodar Krai (Galushko 1980) but was originally misidentified as *S. sisymbriifolium*.

###### Ecology.

Waste ground, roadsides.

###### Residence status.

Casual alien.

###### Pathways of introduction.

The annual species of *S.* sect. *Androceras*, including *S. heterodoxum*, produce abundant seed and have weedy potential (Stern et al. 2010). The old specimen from Russia, which is the only available record so far, lacks sufficient documentation of its locality. For this reason, the pathway of introduction cannot be determined for this species.

##### 8. Solanum rostratum

Taxon classificationPlantaeSolanalesSolanaceae

Dunal, Hist. Nat. Solanum : 234 (1813).

B50C31C6-0A9A-5EAC-BC16-AD33C31D08DA

Fig. 10

###### Type.

France. “In horto Monspeliesi cultum,” without date, *Dunal s.n.*. (holotype: MPU).

**Figure 10.**
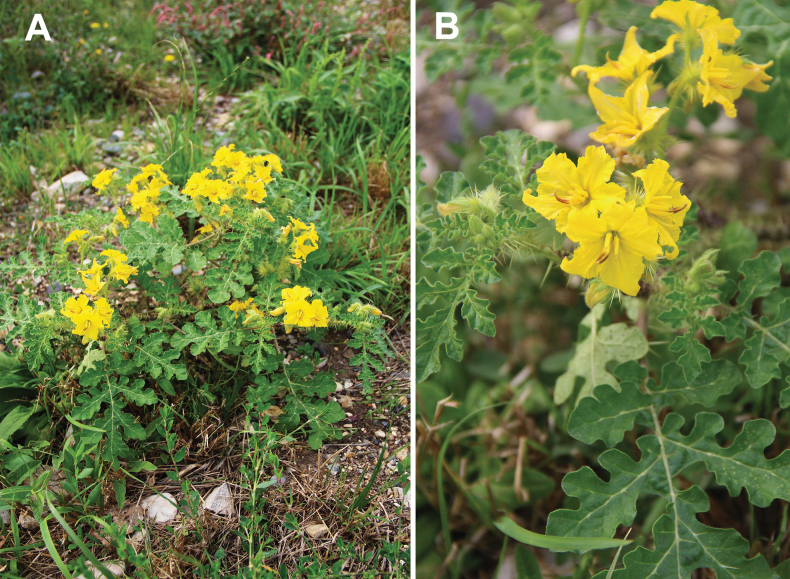
*Solanum rostratum*: **A**. plant in the flowering stage; **B**. close-up of the flowers. Photographer: D. Shilnikov (Neftekumsk, Stavropol’ Krai).

###### Description.

Prickly annual with prostrate or ascending stems up to 150 cm long; prickles basally thickened; indumentum of stellate hairs; leaves green or greyish, pinnatifid with rounded lobes; each cyme of 7–12 flowers; corolla yellow, 25–35 mm in diameter, actinomorphic; one of five anthers longer, 10–14 mm long (all other anthers 6–8 mm long); fruit 9–11 mm in diameter, enclosed in a prickly calyx, dry, irregularly splitting at maturity, sclereidal concretions absent.

###### Taxonomic note.

This species has been previously reported as *Solanum cornutum* auct. non Lam. (Galushko 1980; Poyarkova 1981; Ivanov 2005, 2019; Murtazaliev 2009).

###### Distribution.

This species is native to Mexico and the southern states of the USA. It is alien in other parts of North America, Europe, the Caucasus, southern temperate Asia, northern and southern Africa, and Australia (POWO 2025).

This drought-tolerant North American species was introduced to Europe (France) in the 1920s and has since expanded its range to many regions of the world (Ozuzu et al. 2024, with references therein). In Eastern Europe, the species was first collected in Ukraine in 1928.

In some regions with arid and semi-arid climates, *S. rostratum* is an invasive plant, e.g., in deserts and semi-deserts of China (Jian-Xiao and Yimingniyazi 2023; Huang et al. 2024) and many parts of Australia (Purdie et al. 1982). In contrast, it is considered a rare casual alien, e.g., in Uzbekistan (Sennikov et al. 2018; Khassanov et al. 2020). In the forest zone of Europe and North America, *S. rostratum* is an occasional weed that cannot naturalize in a cold temperate climate (Bassett and Munro 1986; Dzhus 2013).

In Russia, the first collection originated from the Republic of Adygea (North Caucasus) in 1924, although Ulianova (1998) specified the first occurrence in 1918, without citing any specimens or references.

In European Russia (Fig. 7D), *S. rostratum* was mostly found in the southern regions, including the Lower Volga and the plains of the North Caucasus (Poyarkova 1981; Ivanov 2019). The majority of collections date from the 1970s onward.

At the northern limit of its distribution, the species is known from scattered occurrences in Ivanovo Oblast (Borisova 1993, as *S. cornutum*), Saint Petersburg City (Tzvelev 2000), the Mordovian Republic (Silaeva 2010), and the Udmurt Republic (Baranova and Puzyryov 2012).

###### Ecology.

Waste ground, roadsides, and degraded meadows.

###### Residence status.

To date, the species is clearly naturalized in semi-desert plains of European Russia (Kalmyk Republic and probably also in the Volga River delta) and adjacent regions of the North Caucasus (Ubushaev et al. 2011). In other areas of Russia, it is a casual alien.

###### Pathways of introduction.

Transport – Contaminant: seed contaminant.

The species is a grain immigrant in Russia. It was found in waste places at grain-processing factories, at railway stations, and along railways, clearly suggesting its arrival as a grain impurity.

##### 9. Solanum sisymbriifolium

Taxon classificationPlantaeSolanalesSolanaceae

Lam., Tabl. Encycl. 2: 25 (1794).

97FB6457-E01A-5508-910C-2E0342143B9A

Fig. 11

###### Type.

Argentina. Buenos Aires, *P. Commerson s.n*. (lectotype, designated by Vorontsova and Knapp (2016: 307): P-LA [P00357630, lower plant fragment, Morton neg. 8391]; isolectotypes: (P00371604, P00371605, P00371606).

**Figure 11.**
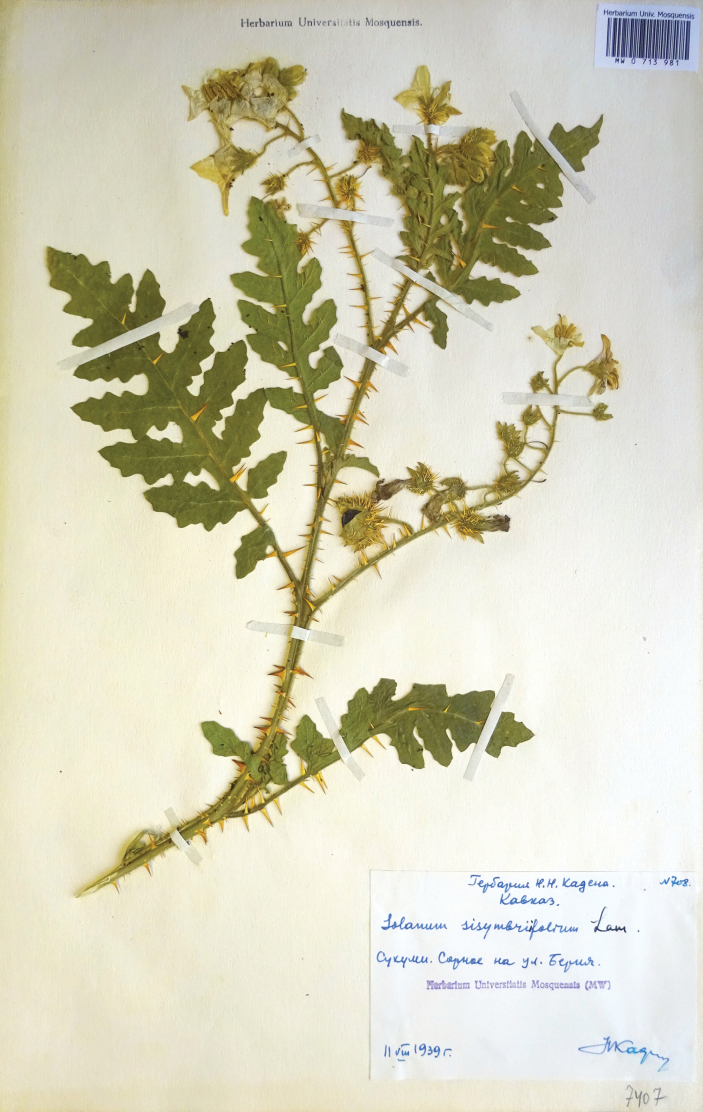
Herbarium specimen of *S. sisymbriifolium* (MW).

###### Description.

Prickly and sticky annual; prickles basally slightly thickened; indumentum of glandular hairs mixed with stellate and simple hairs; leaves pinnatisect or bipinnatisect, leaf lobes acute; corolla white or violet, 20.0–30.0 mm in diameter, actinomorphic; stamens equal, 10.0–15.0 mm long; fruit 10–20 mm in diameter, red, exserted.

###### Taxonomic note.

The species reports from Krasnodar Krai, North Caucasus (Galushko 1980), are misidentifications corresponding to *S. heterodoxum*.

###### Distribution.

This South American species was introduced very early into European botanical gardens (ergasiophyte) and, in addition, was planted in fallow potato fields as a trap crop for plant-parasitic nematodes (Aubriot and Knapp 2022). Outside its native range, it is considered an aggressive weed in tropical and subtropical parts of Asia (Zhang et al. 1994; Saha and Datta 2013), Africa, and Australia (Jaeger 1985), as well as in Italy (https://dryades.units.it/floritaly/index.php?procedure=taxon_page&tipo=all&id=4719).

It can also thrive in a subtropical climate in the Black Sea Region, e.g., in the Republic of Abkhazia, where it was first collected in 1952 (Kolakovsky 1986), with many subsequent records dating back to the 1960s (LE!, MW!). *Solanum sisymbriifolium* appears to be naturalized in Abkhazia, where it is found mainly in coastal sands and ruderal places. Based on these findings, new records from neighboring Krasnodar Krai (North Caucasus), especially in coastal areas, are highly likely.

**Figure 12.**
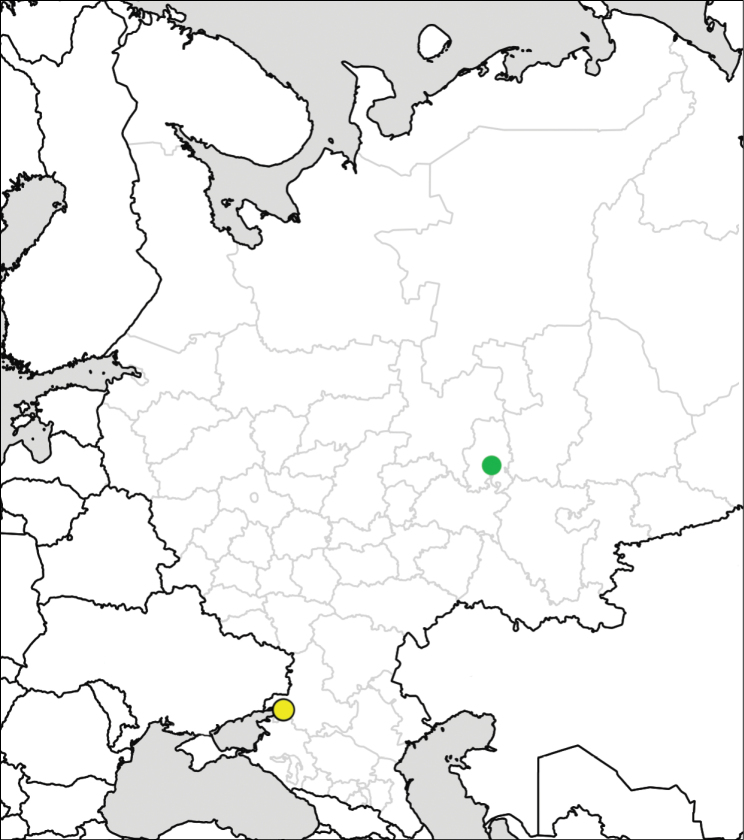
Records of *Solanum sisymbriifolium* in European Russia. Yellow dot denotes an old record from the late 19^th^ century that has not been confirmed by recent observations.

In European Russia, the species was found only twice in the steppe and forest-steppe zones (Fig. 12). Its oldest record (a specimen at MW, collected without precise locality information by I.F. Kramsakov in the 1880s) originated from Taganrog or Novocherkassk in Rostov Oblast. The second specimen was collected from a railway in Izhevsk, Udmurt Republic (Tuganaev and Puzyryov 1988; Baranova and Puzyryov 2012).

###### Ecology.

Railways, waste places.

###### Residence status.

Casual alien.

###### Pathways of introduction.

Transport – Contaminant: seed contaminant.

As the species was collected from railways, we assume its arrival with contaminated grain.

## Discussion

Fruit and seed characters of *Solanum* are seldom discussed in the Russian literature. For this reason, we provide more data on carpological features, especially seed number and the presence of the so-called stone cells, as well as on dispersal facilities. Fruit and seed characters can provide additional information when the leaves are already withered.

The typical *Solanum* fruit is a berry that can be fleshy or dry. In contrast, *Solanum rostratum* has dry fruits that change in color from green to black or dark brown when ripe (Yang et al. 2019). Its fruits are surrounded by a persistent prickly calyx, which may enhance epizoochorous dispersal. Dry fruits of this species can rupture irregularly and release the seeds, which are further dispersed by wind, rain, and other abiotic factors (Symon 1984, with references therein). Other alien species found in European Russia have fleshy berries that can be black (*S. emulans*, *S. scabrum*), green or olive-green (*S. nitidibaccatum*, *S. triflorum*), red (*S. sisymbriifolium*), or yellow or orange (*S. villosum*). Ornithochory is a likely dispersal mode for species with fleshy fruits (Symon 1979; Knapp 2002). Fruits can also be eaten by mammals (theriochory), e.g., in *S. sarrachoides* (Tamboia et al. 1996), if they have low glycoalkaloid content, as in *S. americanum* (Cipollini and Levey 1997). Hydrochory is also indicated in the literature, e.g., in *S. sarrachoides* (del Monte and Sobrino 1993). Furthermore, rolling of spherical fruits is a typical case of autochory.

Fruit diameter varies from 10 to 20 mm in *S. scabrum* and *S. sisymbriifolium*. A smaller diameter (9–13 mm) is typical for *S. rostratum* and *S. triflorum*. Other studied species usually have fruits 4–8 mm in diameter.

All *Solanum* species have more than ten seeds per fruit. In some cases, seed number is used as a diagnostic character. Militzer (1964) indicated that the fruit of *S. nitidibaccatum* contains up to 16 seeds, compared with those of *S. nigrum* (20+). Nevertheless, the tiny forms of *S. nigrum* growing in Moscow Oblast and studied by us have 15–18 seeds. This observation agrees with a detailed study by Ogg et al. (1981), who counted 15 to 60 seeds per fruit in *S. nigrum*. A fruit of *S. nitidibaccatum* from a plant collected in the Republic of Mordovia had 22 seeds, which falls within the range (13–24) indicated by Knapp et al. (2023). A morphologically similar species, *S. sarrachoides*, has more numerous seeds (24–93) per fruit. Comparisons of some species (*S. nigrum*, *S. scabrum*, *S. sisymbriifolium*, *S. triflorum*), among others, are also referenced in Symon (1987), who revealed the following ranges of seed numbers: (78–)129(–163) seeds per fruit in *S. triflorum*, (33–)116(–205) in *S. sisymbriifolium*, (79–)103(–126) in *S. scabrum*, and (10–)37(–72) in *S. nigrum*. Based on these results, it can be concluded that seed number is very variable but can be used to differentiate similar-looking species, e.g., *S. nitidibaccatum* and *S. sarrachoides*.

Along with seeds, the fruits of many *Solanum* species also contain sclerotic granules (sometimes referred to as “stone cells”). These small inclusions are sclereidal concretions in the pericarp that have been interpreted as rudiments of a drupaceous fruit type (Danert 1969). They can be completely absent in the fruits of some species, e.g., in the prickly *S. rostratum* and *S. sisymbriifolium* (Symon 1987). The absence of sclereidal concretions has been reported in *S. nigrum* (Ogg et al. 1981; Symon 1987), *S. scabrum* (Symon 1987), and *S. villosum* (Särkinen et al. 2018), whereas such inclusions are present in other species of the Morelloid clade. Their number varies: 1–3 per fruit in *S. nitidibaccatum* (Särkinen et al. 2018; confirmed by the present study), 4–6 in *S. sarrachoides* (Knapp et al. 2023), 6–10 in *S. emulans* (Knapp et al. 2019), and 2–8 in *S. americanum* (Symon 1987). The fruits of *S. triflorum* contain numerous (20–35) concretions (Symon 1987).

For reliable identification within certain species pairs (e.g., *S. nigrum* vs. *S. villosum*, *S. nitidibaccatum* vs. *S. sarrachoides*), plants should be collected with ripe fruits.

## Conclusion

The number of alien species of *Solanum* in European Russia and the North Caucasus is much higher than indicated in previous major floristic treatments (e.g., Poyarkova 1981; Tzvelev 2000; Ivanov 2019; see Table 1).

Out of the ten species included in the keys, nine taxa are neophytes, and only *S. nigrum* is an archeophyte mentioned in the oldest checklists for the flora of European Russia and the North Caucasus (e.g., Sobolewski 1799; von Bieberstein 1808; von Ledebour 1849). Among the neophytes, *S. scabrum* is an intentionally introduced species that rarely escapes from cultivation and is mostly found in landfills. All other taxa were brought without specific intent and should be referred to as xenophytes; when their pathway of introduction is known, it indicates their arrival with contaminated grain imported from North America. All nine neophytes were collected mostly in ruderal or disturbed places, but *S. triflorum* is capable of invading natural and partially degraded sandy steppes. *Solanum nitidibaccatum* occupies both ruderal and segetal habitats, whereas other species are typically ruderal.

Most of the species are casual aliens, but *S. nitidibaccatum*, *S. rostratum*, *S. triflorum*, and *S. villosum* are clearly established in some parts of the studied area. Their further expansion is expected.

## Supplementary Material

XML Treatment for Solanum emulans

XML Treatment for Solanum nitidibaccatum

XML Treatment for Solanum scabrum

XML Treatment for Solanum triflorum

XML Treatment for Solanum villosum

XML Treatment for Solanum elaeagnifolium

XML Treatment for Solanum heterodoxum

XML Treatment for Solanum rostratum

XML Treatment for Solanum sisymbriifolium

## Additional information

### Use of AI

No use of AI was reported.

### Author ORCIDs

Alexander P. Sukhorukov https://orcid.org/0000-0003-2220-826X

Sandra Knapp https://orcid.org/0000-0001-7698-3945

Maria Kushunina https://orcid.org/0000-0003-2541-6494

Alexander N. Sennikov https://orcid.org/0000-0001-6664-7657

### Data availability

The dataset associated with this study has been deposited in an external repository and is publicly accessible. The dataset can be accessed at GBIF via the following link: https://doi.org/10.15468/pgceqx.
